# Cytomegalovirus‐induced oral ulcers: A case report and literature review

**DOI:** 10.1002/ccr3.7459

**Published:** 2023-06-07

**Authors:** Margaux Pinana, Camille Rapoport, Nicolas Champtiaux, Géraldine Lescaille, Yves Allenbach, Juliette Rochefort

**Affiliations:** ^1^ Department of Oral Mucosal Pathology, AP‐HP Groupe Hospitalier Pitié‐Salpêtrière University of Paris Paris France; ^2^ Department of Anatomopathology, AP‐HP Groupe Hospitalier Pitié‐Salpêtrière Sorbonne University Paris France; ^3^ Department of Internal Medicine Groupe Hospitalier Pitié‐Salpêtrière Paris France

**Keywords:** cytomegalovirus, immunodepression, infection, systemic lupus erythematosus

## Abstract

Cytomegalovirus (CMV) ulcerations are rare clinical entities, but their occurrence is favored in immunocompromised patients who present a favorable environment for opportunistic infections. We describe the case of a patient treated for a systemic lupus erythematosus suffering from deep oral ulcerations. The case illustrates the complexity of establishing a precise etiological diagnosis of CMV lesions, as the diagnostic hypothesis can be varied: related to an immunodeficiency disorder or drug‐induced toxidermia.

## 1 INTRODUCTION

Human cytomegalovirus (CMV) or Human Herpes Viridae 5 (HHV5), belonging to the Herpesviridae family, is a widely distributed virus and around 60% of the world's population has already been exposed (presence of anti‐CMV antibodies). It is an opportunistic virus that can be transmitted via saliva, sexual secretions, breastfeeding, and blood transfusions. In immunocompetent patients, primary infection is usually asymptomatic, but can occasionally cause a mononucleosis‐like syndrome. Clinical manifestations of CMV infection are various with potential pulmonary, gastrointestinal, or hepatic implications as well as mucocutaneous manifestations such as ulcerations.^1^, ^2^

Immunodepression is a breeding ground for opportunistic infections, especially for CMV infection or its reactivation.^3^ The scientific literature frequently reports the association between oral ulcerations and immunocompromised backgrounds favorable to opportunistic infections such as CMV. Among the general pathologies inducing a state of immunodepression, systemic lupus erythematosus (SLE) is an autoimmune disease, which can cause mucosal damages. It is frequently accompanied by leukopenia and is treated with corticosteroids, immunosuppressors, biotherapies, and/or hydroxychloroquine. Patients with SLE present a significant risk of infection due to both disease and its treatment and are therefore susceptible to opportunistic infections.^4^ This article illustrates the case of CMV oral ulcerations in a patient treated for SLE.

## 2 CASE REPORT

**FIGURE 1:**
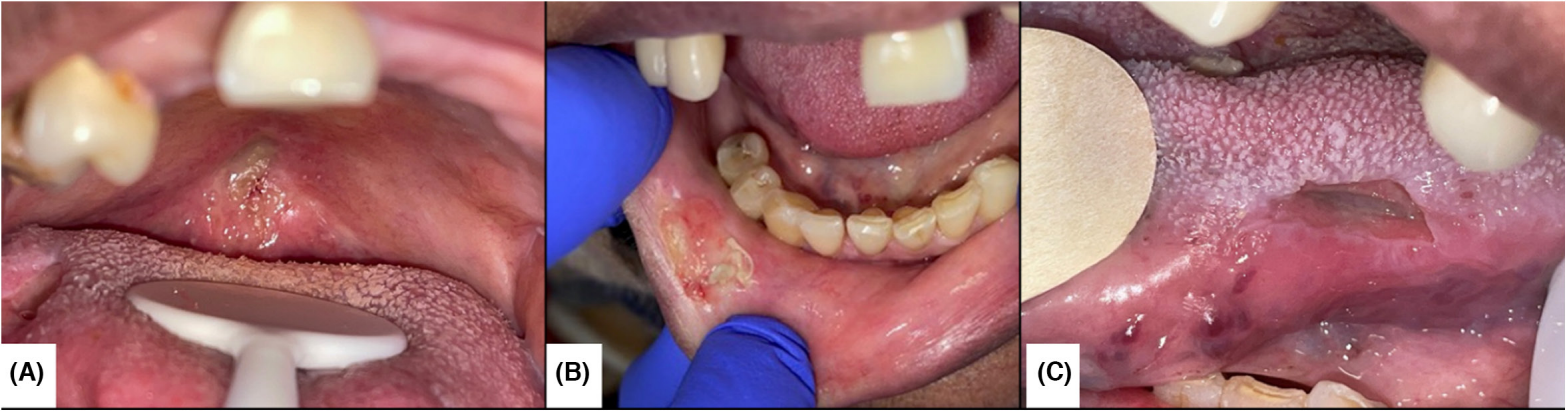
Intra‐oral views of soft palate (A), lower lip (B) and left‐side tongue (C) ulcerations.

A 62‐year‐old female patient hospitalized for the deterioration of her general state was suffering from a systemic lupus erythematosus (SLE), diagnosed 4 months earlier. She had skin, neurological, pulmonary, and renal implications, treated with mycophenolic acid (Cellcept), hydroxychloroquine, and corticosteroid therapy. She suffered from oral ulcerations for 2 weeks, causing dysphagia and dehydration. These ulcerations were multiple, painful, and deep, located on the mucosal side of the lower lip, soft palate, and left side of the tongue. These lesions all had a “die‐cut” appearance with well‐defined edges and extended deep in the tissue (Figure 1A–C).

**FIGURE 2:**
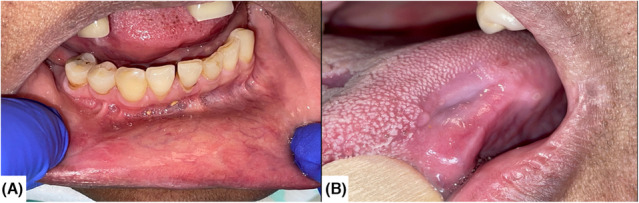
Intra‐oral views of cicatrization after antivirals treatment on lower lip (A) and left‐side tongue (B).

Her blood count showed a lymphopenia (0.26 × 10^9^/L). Further examinations did not reveal the involvement of any other organ. Viral samples (CMV, HHV1,2 and 5) were taken, a biopsy was carried out, and local therapies were implemented. Standard histological analysis reported ulceration with viral cytopathogenic effects (Koilocytes). There were clear intracytoplasmic inclusions and rare basophilic nuclear inclusions. Immunohistochemical staining realized on the biopsy sample showed the presence of CMV, and a blood PCR was positive in high quantity for CMV (×5log). The diagnostic hypotheses were oral lupus manifestations, infectious ulcerations due to immunosuppression, or toxidermia related to recently introduced drugs, in particular Cellcept. The patient was treated with antivirals, Ganciclovir (10 mg/kg/24 h intravenous) for 2 weeks and then with Valganciclovir (900 mg/24 h) for 4 weeks. Cellcept was suspended while the ulcerations improved, and a pharmacovigilance investigation was conducted regarding its possible involvement. A decrease in symptomatology was noted after 1 week of treatment. The oral ulcers healed within 2 weeks. Now, at 18 months, the patient does not show any sign of recurrence (Figure 2A,B).

## 3 DISCUSSION

Ulcerations are nonspecific lesions characterized by deep substance loss frequently found in immunocompromised patients. The etiology is various: ulcerations due to drug‐induced aplasia in the case of antirejection therapy (organ transplant), immunosuppressive drugs, chemotherapy, autoimmune diseases (SLE, rheumatoid arthritis, multiple sclerosis, etc.), or by congenital or acquired immune deficiency.^1^, ^2^, ^3^ Cases of CMV‐positive oral ulcerations were most commonly reported in the literature in patients with human immunodeficiency virus (HIV) in AIDS stage. However, in recent years, due to medical and therapeutic advances and the very low number of patients reaching the AIDS stage, the cases of CMV ulceration reported in these patients are now very rare, and more often found in patients undergoing immunosuppressive therapy for organ transplant, as shown in Table 1.

**TABLE 1:** CMV‐induced oral ulcers in immunocompromised patients: a review of literature.

Authors	Number of case	Gender	Age	Localization	Disease	Treatment(s)
Kaisar MO et al. (2008)^5^	2	F	62	Single tongue ulceration	Kidney transplant	Valganciclovir
		M	34	Deep tongue ulceration	Kidney tranplant	Ganciclovir (IV)
Lopez‐Pintor RM et al. (2009)^6^	6	4F 2M	30–66	Soft (2) and hard palate (2), gingiva (4), tongue (1), floor of the mouth (2)	Kidney transplant	Ganciclovir (IV)
Planas‐Ciudad S et al. (2017)^7^	1	M	40	Single ulceration of the mucosal side of the lower lip	Heart transplant	Ganciclovir
Mainville GN et al., 2015^8^	1	F	46	Single ulceration: dorsal side of the tongue, median	Kidney and pancreas tansplants	Gancyclovir, Valganciclovir
Huang S et al. (2019)^9^	1	M	65	Single ulceration on the vestibular side of the gingiva (teeth 33/34)	Biotherapy (Wegener's disease)	Ganciclovir
Yen H et al. (2022)^10^	1	M	70	Multiple lip and tongue ulcerations	Anti‐PDL1 chemotherapy	Ganciclovir
Faram et al. (2022)^11^	1	F	18	Widespread oral infected ulcer	Systemic lupus erythematosus	

*Abbreviations*: F, female, M, male, IV, intraveno.

Cytomegalovirus infection is manifested by nonspecific symptoms such as fever, asthenia, and more rarely by dermatological involvement, of which ulceration is one of the main manifestations.^1^, ^12^ In the case of mucosal damage, it presents a “die‐cut” appearance with well‐defined edges and extending deep in the tissue.^13^ CMV‐induced oral ulcers are uncommon entities mostly found in patients with a high risk of infection, whether a primary infection or the reactivation of a latent virus. They can be localized on the entire oral mucosa with a prevalence for the palate (hard and soft), single or multiple, often deep and painful, with raised and erythematous edges.^14^ The diagnosis of CMV ulceration is based on viral‐DNA detection with a blood PCR (polymerase chain reaction) or immunohistochemical examination by biopsy, searching for CMV antibodies. Anatomopathological examination reveals ulceration characterized by multiple intracytoplasmic inclusions and nucleoli surrounded by a clear halo, presenting a typical “owl's eye” appearance.^8^

Several authors have investigated the association of SLE with concomitant CMV infection, which occurs in approximately 10% of patients with SLE and may take the form of CMV skin ulcerations.^3^, ^12^, ^15^, ^16^ However, descriptions of cases of oral CMV ulcerations in SLE patients are very rare in the scientific literature (Table 1). One paper has reported a case of CMV infected oral ulcers in a woman with SLE, with no distinguishing clinical features.^11^ Authors have reported that CMV infection occurs mainly during the active phases of lupus (primary or recurrent CMV infection), promotes an exacerbation of lupus symptoms, and can sometimes be the cause of disease onset.^17^, ^18^ This situation is explained by the ability of the virus to induce the expression of nuclear antigens involved in the autoimmune mechanism of the disease.^19^ Other authors also highlighted gastrointestinal, hepatic, and pulmonary infectious complications related to CMV infection in lupus patients, without reporting specific oral manifestations.^8^, ^19^, ^20^

There are specific mucocutaneous manifestations of SLE, with malar rash or erythematous and erosive aspects called discoid lesions in the oral cavity, and also nonspecific lesions.^21^, ^22^ Systemic lupus erythematosus may be presents with oral involvement, including ulcerations, without concomitant viral infection. They are usually found on the palate, cheeks, or tongue and are sometimes the reason for the discovery of an underlying lupus disease, especially in children.^21^ They do not have the same typical appearance of CMV‐induced oral ulceration with a so‐called “die cut” shape, as described in our clinical case.

Identifying the etiology of these ulcerations represents a real challenge for clinicians. They may be the oral expression of underlying lupus disease, or the manifestation of opportunistic infections due to immunosuppression induced by immune dysfunction related to SLE, to treatments, or to toxidermia related to drugs.^23^ In the clinical case reported, pharmacovigilance investigation established that mycophenolic acid and long‐term corticosteroid therapy, which certainly induced immunodepression, were not the cause of the ulcerations. The etiology retained for these CMV ulcerations was drug‐induced lymphopenia. Oral CMV ulcerations are the local expression of a viral infection, but the general repercussion could be important, with the risk of damage to other organs and possibly severe decompensation. Antivirals (Aciclovir, Ganciclovir, and Valganciclovir) are the treatment of choice for CMV infection, regardless of its clinical manifestation, while local care may be associated with the case of oral ulcerations for analgesic purposes.^24^

## 4 CONCLUSION

The case reported here illustrates the importance of clinical investigation and the prescription of targeted complementary examinations, which are essential for the management of these lesions. Although rare, oral cavity specialists should be aware of this clinical entity of CMV‐induced oral ulceration in order to identify severe immunosuppression situations as early as possible.

## AUTHOR CONTRIBUTIONS

**Margaux Pinana:** Investigation; writing – review and editing. **Camille Rapoport:** Validation. **Nicolas Champtiaux:** Validation. **Géraldine Lescaille:** Validation. **Yves Allenbach:** Validation. **Juliette Rochefort:** Investigation; supervision; validation; writing – review and editing.

## FUNDING INFORMATION

No financial support was received.

## CONFLICT OF INTEREST STATEMENT

The authors declare no conflicts of interest.

## CONSENT

Written informed consent was obtained from the patient to publish this report in accordance with the journal's patient consent policy.

## Data Availability

The data that support the findings of this study are available upon request from the corresponding author.
